# A bloody interaction: plasma proteomics reveals gilthead sea bream (*Sparus aurata*) impairment caused by *Sparicotyle chrysophrii*

**DOI:** 10.1186/s13071-022-05441-1

**Published:** 2022-09-10

**Authors:** Enrique Riera-Ferrer, M. Carla Piazzon, Raquel Del Pozo, Oswaldo Palenzuela, Itziar Estensoro, Ariadna Sitjà-Bobadilla

**Affiliations:** Fish Pathology Group, Department of Marine Species Biology, Culture and Pathology, Institute of Aquaculture Torre de La Sal–Consejo Superior de Investigaciones Científicas (IATS–CSIC), Ribera de Cabanes, 12595 Castellón Spain

**Keywords:** Monogenea, SWATH-MS, Haemoglobin, Haemostasis, Immune system, Host-parasite interactions, Cholesterol

## Abstract

**Background:**

Sparicotylosis is an enzootic parasitic disease that is well established across the Mediterranean Sea. It is caused by the polyopisthocotylean monogenean *Sparicotyle chrysophrii* and affects the gills of gilthead sea bream (GSB; *Sparus aurata*). Current disease management, mitigation and treatment strategies are limited against sparicotylosis. To successfully develop more efficient therapeutic strategies against this disease, understanding which molecular mechanisms and metabolic pathways are altered in the host is critical. This study aims to elucidate how *S. chrysophrii* infection modulates the plasma proteome of GSB and to identify the main altered biological processes involved.

**Methods:**

Experimental infections were conducted in a recirculating aquaculture system (RAS) in which naïve recipient GSB ([R]; 70 g; *n* = 50) were exposed to effluent water from *S. chrysophrii*-infected GSB (98 g; *n* = 50). An additional tank containing unexposed naïve fish (control [C]; 70 g; *n* = 50) was maintained in parallel, but with the open water flow disconnected from the RAS. Haematological and infection parameters from sampled C and R fish were recorded for 10 weeks. Plasma samples from R fish were categorised into three different groups according to their infection intensity, which was based on the number of worms fish^−1^: low (L: 1–50), medium (51–100) and high (H: > 100). Five plasma samples from each category and five C samples were selected and subjected to a SWATH-MS proteome analysis. Additional assays on haemoglobin, cholesterol and the lytic activity of the alternative complement pathway were performed to validate the proteome analysis findings.

**Results:**

The discriminant analysis of plasma protein abundance revealed a clear separation into three groups (H, M/L and C). A pathway analysis was performed with the differentially quantified proteins, indicating that the parasitic infection mainly affected pathways related to haemostasis, the immune system and lipid metabolism and transport. Twenty-two proteins were significantly correlated with infection intensity, highlighting the importance of apolipoproteins, globins and complement component 3. Validation assays of blood and plasma (haemoglobin, cholesterol and lytic activity of alternative complement pathway) confirmed these correlations.

**Conclusions:**

Sparicotylosis profoundly alters the haemostasis, the innate immune system and the lipid metabolism and transport in GSB. This study gives a crucial global overview of the pathogenesis of sparicotylosis and highlights new targets for further research.

**Graphical Abstract:**

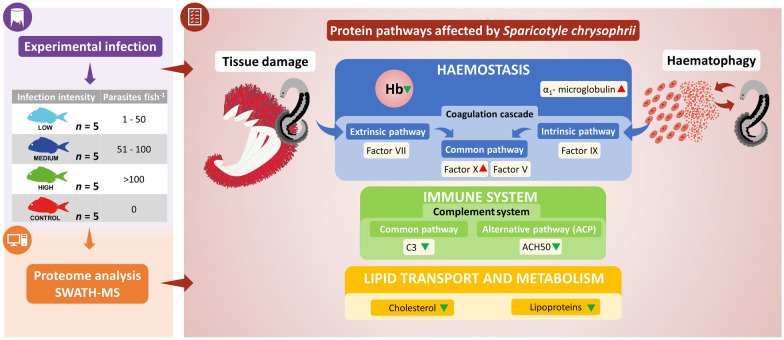

**Supplementary Information:**

The online version contains supplementary material available at 10.1186/s13071-022-05441-1.

## Background

Sparicotylosis is a gill infection caused by the polyopisthocotylean monogenean parasite *Sparicotyle chrysophrii* (formerly* Microcotyle chrysophrii*; Microcotylidae). This parasite has a direct life-cycle in which gravid adult specimens shed embryonated eggs into the water column from which motile ciliated larvae hatch after 5–10 days. If any hosts are nearby, the larvae will attach to their gill filaments, giving rise first to post-larvae and subsequently to juveniles and adults [1, 2]. This disease is enzootic in the Mediterranean Sea, affecting several fish species of the Sparidae family [3–7], amongst which gilthead sea bream (GSB; *Sparus aurata*) stands out as the type host and its commercial relevance in the Mediterranean aquaculture industry [8].

Sparicotylosis is associated with lethargy due to hypoxia, severe anaemia and emaciation. Gill histopathological findings, such as lamellar synechiae, clubbing and shortening, epithelial hyperplasia resulting in secondary lamellae fusion and proliferation of chloride cells, have been described [9, 10].

Disease management in on-growing offshore net pens is a complex process. The high stocking densities, the proximity of the cages, the marine currents and the seeding of fingerlings without year-class separation or fallowing strategies create a perfect niche for amplification and dissemination of any pathogen. Current methods to control sparicotylosis rely on disinfectant bath treatments, net changing or cleaning [11] and nutraceutical formulation feedings [12].

For more than two decades, efforts have been made to widen the chemotherapeutic alternatives against *S. chrysophrii* [9, 12, 13], but only hydrogen peroxide and formalin baths, which present a narrow therapeutic index and several concerns [12, 14–17], remain as treatment options against sparicotylosis. The successful development of more efficient therapeutic strategies to control sparicotylosis critically relies on knowledge of both the host’s and parasite’s molecular mechanisms and metabolic pathways, which are relevant in the host-parasite relationship. Thus far, few studies dealing with *S. chrysophrii*—GSB interactions have been published. Henry et al. [18] described the inhibition of the humoral response and activation of cellular components in GSB—*S. chrysophrii* long-term infections. A subsequent tissue-level transcriptomic analysis of mild *S. chrysophrii* infections revealed that apoptosis, inflammation and cell proliferation played leading roles in the gills, whereas a hypometabolic response was detected in the spleen [19].

In recent years, proteomic analyses have transformed how host-parasite interactions are studied and understood. These interactions can be studied either by determining the expression of the parasite proteome throughout the infection process (i.e. tegumental and secreted proteins and extracellular vesicles), by detecting parasite proteins in its host, or by defining the infection effects on the host’s proteome. Thus far, significant progress has been achieved in understanding critical high-impact zoonotic and animal parasitic diseases through this technology [20–29].

The aim of the study reported here is to elucidate how *S. chrysophrii* infection modulates the plasma proteome of GSB and to identify the main altered biological processes involved.

## Methods

### Animals, experimental infections and sampling

Healthy GSB juveniles were purchased from a Mediterranean-based hatchery (Piscimar, Burriana, Spain) and maintained at the indoor experimental facilities of the Institute of Aquaculture Torre de La Sal–Consejo Superior de Investigaciones Científicas (IATS–CSIC; 40°5′N, 0°10′E) under natural photoperiod and temperature conditions. Water parameters were monitored; oxygen saturation was kept > 85% and unionised ammonia was kept < 0.02 mg l^−1^ in all tanks. All animals used in this experiment were fed twice daily, 5 days per week, until visual satiety, with a commercial dry pellet diet of the adequate size.

The experimental infection was conducted in a recirculation aquaculture system (RAS) from April to June, with water temperatures ranging from 15.23 °C to 23.85 °C. The experimental design consisted of a recipient (R) tank (200 l) holding naïve GSB (70 g; *n* = 50) receiving water from a donor (D) tank (200 l) with *Sparicotyle chrysophrii*-infected GSB (98 g; *n* = 50). The infective status of D fish was confirmed by observation of *S. chrysophrii* eggs on egg collectors placed in the D tank. In parallel, an additional tank with control (C); *n* = 50) unexposed naïve fish from the same stock was maintained with the open water flow disconnected from RAS but the same temperature and oxygen conditions maintained.

After the beginning of the exposure to *S. chrysophrii*, one sampling was performed every 2 weeks for 10 weeks. Three casualties were registered in the R group, one at day 44 and two at day 49 post exposure. In each sampling, 20 fish (10 R, 10 C) were euthanised by tricaine methanesulfonate (MS-222) overdose (0.1 g l^−1^), and blood was collected from the caudal vein using heparinised syringes, taking special care to avoid haemolysis; the collected blood samples were constantly maintained on ice. Details on fish numbers, sampling times and the downstream use of individual samples are indicated in Table 1 and Additional file 1: Figure S1. Following blood collection, haemoglobin (Hb) values were immediately recorded (HemoCue® 201+ Hb System; HemoCue AB, Ängelholm, Sweden). The remaining blood was centrifuged at 3000 *g* for 30 min, and plasma was stored at − 80 °C until processing. The right-side gill arches of each R specimen were dissected and* S. chrysophrii* counts of adult and juvenile specimens were carried out in situ under a stereomicroscope to determine the infection intensity. Infection intensities were extrapolated for the eight gill arches of each fish after Riera-Ferrer et al. [30].

**Table 1:** Experimental setup and sampling details

Sampling (S)	Days post exposure (*dpe*)	Mean temperature (°C)	Sampled fish	Mean infection intensity in R fish (± SEM)^a^	Application				
					Proteome analysis	Haemoglobin analysis	Cholesterol assay	Alternative complement pathway	Biotin assay
S1	14	17.88	10C; 10R	50.4 ± 15.32	4R	10C; 10R	10C; 10R	10C; 10R	10C; 10R
S2	28	20.58	10C; 10R	134.4 ± 22.77	1R	10C; 10R	10C; 10R	10C; 10R	10C; 10R
S3	42	20.48	10C; 10R	117 ± 32.89	6R	10C; 10R	10C; 10R	10C; 10R	10C; 10R
S4	57	22.02	10C; 10R	76.8 ± 15.30	2C; 3R	10C; 10R	10C; 10R	10C; 10R	10C; 10R
S5	68	23.35	10C; 7R	53.8 ± 16.32	3C; 1R	10C; 7R	10C; 7R	10C; 7R	10C; 7R

*C* Control (unexposed) fish,* R* recipient (naïve) fish,* SEM* standard error of the mean

^a^Mean intensity of infection for 8 gill arches determined based on number of worms fish^−1^

### Ethics statement

All experiments were carried out according to current Spanish (Royal Decree RD53/2013) and EU (2010/63/EU) legislation on the handling of experimental fish. All procedures were approved by the Ethics and Animal Welfare Committee of the Institute of Aquaculture Torre de la Sal (IATS—CSIC, Castellón, Spain), CSIC and “Generalitat Valenciana” (permit number 2018/VSC/PEA/0240).

### Plasma proteome analysis

#### Candidate selection

A total of 20 different plasma samples were processed for proteomic analysis by the Central Service for Experimental Research (SCSIE) proteomics facility at the University of Valencia, Spain, which is a member of the Spanish network of proteomic research facilities (ProteoRed). All R fish were categorised into three groups according to their infection intensity recorded as worms fish^−1^: low, medium and high (L: 1–50; M: 51–100; H: > 100 worms fish^−1^,respectively). Five plasma samples of each category and five C samples were selected for testing of infection intensity. The remaining plasma samples were stored until used in the validation assays (alternative complement pathway activity, cholesterol and biotin concentration).

#### Sample preparation

For the albumin depletion assay, 12 µl of each individual sample was precipitated with cold ethanol at a final concentration of 40% (v/v). The precipitation was incubated overnight at 5 °C and then centrifuged at 15,000 *g* for 1 h. The albumin-containing supernatant was then removed, and the pellets air-dried. The pellets were then dissolved in 50 μl of 0.5% sodium dodecyl sulfate (SDS) in 50 mM ammonium bicarbonate. The proteins were quantified with a protein quantification assay kit (Macherey−Nagel GmbH & Co. KG, Düren, Germany) according to the manufacturer’s instructions.

Due to the presence of lipids in the samples, 7 µg of protein was loaded in a one-dimensional (1D) polyacrylamide gel electrophoresis (PAGE) system without resolving and in-gel digested. The gel slices of each sample were cut into small cubes and sequentially dehydrated with 50% acetonitrile in 50 mM ammonium bicarbonate and 100% acetonitrile. Cysteine residues were reduced by 10 mM dithiothreitol in 50 mM ammonium bicarbonate buffer at 60 °C for 20 min, and sulfhydryl groups were alkylated with 5.5 mM iodoacetamide in 50 mM ammonium bicarbonate, in the dark, at room temperature, for 30 min. Gel cubes were incubated overnight at 37 °C in 100 μl of 50 mM ammonium bicarbonate solution with 400 ng of trypsin. The digestions were quenched with trifluoroacetic acid (final concentration: 1%). The supernatants were then removed, and the gel plugs were dehydrated with neat acetonitrile. The acetonitrile peptide solutions were recombined with the previous supernatants. The digestion mixture was dried in a vacuum centrifuge and resuspended in 20 μl of 2% acetonitrile, 0.1% trifluoroacetic acid.

#### Sequential window acquisition of all theoretical mass spectra analysis

For every mixture of digested peptide, 2 µl of peptide mixture sample was loaded in a NanoLC 425 HPLC system (Eksigent Technologies, Redwood, CA, US) onto a trap column (C18-CL 3 μm, 300 Å; internal diameter [id]: 350 μm × 0.5 mm) and desalted with 0.1% trifluoroacetic acid at a flow rate of 5 µl min^−1^ for 5 min. The peptides were then loaded onto an analytical column (C18-CL 3 μm, 120 Ᾰ; id: 75 μm × 150 mm; Eksigent Technologies) equilibrated in 5% acetonitrile, 0.1% formic acid. Peptide elution was carried out with a linear gradient of 7–40% acetonitrile with 0.1% formic acid at a flow rate of 300 nl min^−1^. Peptides were analysed in a nanoESI qQTOF mass spectrometer (TripleTOF 6600+ System; AB Sciex, Framingham, MA, USA). The samples were ionised in a Source Type: Optiflow < 1 µl Nano applying 3.0 kV to the spray emitter at 200 °C. The TripleTOF mass spectrometer was operated in swath mode, in which a 0.050-s time-of-flight mass spectrometry (TOF–MS) scan from 350 to 1250* m/z* was performed. Thereafter, 0.080-s product ion scans were acquired in 100 variable windows from 400 to 1250 *m/z*. The total cycle time was 2.79 s. The individual sequential window acquisition of all theoretical mass spectra (SWATH-MS) injections were randomised to avoid bias in the analysis. Prior to running the individual samples, a pooled sample was injected to determine the best gradient and sample amount.

#### Spectral library building

Plasma aliquots of all samples were pooled to build the spectral library by in-gel digestion and liquid chromatography-tandem mass spectrometry (LC–MS/MS) with data-dependent acquisition (DDA) in order to separate and identify the proteins present in the samples. After resolving the 1D SDS-PAGE, the gel lane corresponding to the library was cut into pieces, and each piece was digested with trypsin, extracted with acetonitrile, dried and resuspended as described above. Exactly as described before, each library sample was first loaded into a trap column and then into an analytical column, before loading the eluted peptides in the nanoESI qTOF mass spectrometer for analysis in DDA mode. Survey MS1 scans were acquired from 350 to 1400 *m/z* for 250 ms. The quadrupole resolution was set to ‘low’ for MS2 experiments, which were acquired at 100–1500 *m/z* for 25 ms in ‘high sensitivity’ mode. The following switch criteria were used: charge: 2+ to 4+ ; minimum intensity; 100 counts per second (cps). Up to 100 ions were selected for fragmentation after each survey scan. Dynamic exclusion was set to 15 s.

The obtained DDA data files were processed using the ProteinPilot v5.0 (AB Sciex) search engine, and a single list of peaks was generated using the default parameters and combining the acquired information of all gel fragments. The Paragon algorithm (ProteinPilot software; AB Sciex) was used to search against 279,921 sequences available in GSB protein databases (NCBI, UniProt and transcriptome from the genome assembly [31]). A false discovery rate (FDR) correction was applied for the validation of the data. The identified proteins were grouped based on MS/MS spectra by the ProteinPilot Pro Group algorithm to avoid using the same spectral evidence for more than one protein. The protein within each group that could explain more spectral data with a 95% confidence threshold was depicted as the primary protein of the group. To increase the spectral data with data-independent acquisition (DIA) information, the data from the pooled samples were analysed by DIA Umpire as previously published [32].

#### Protein quantification

The SCIEX.wiff data-files obtained from SWATH runs of the 20 individual plasma samples were loaded into PeakView v2.1 (AB Sciex) with the generated spectral library consisting of a combination of data-dependent and -independent acquisition information, obtained from the pooled sample interrogated in the available protein databases at a peptide confidence threshold of 95% and an FDR < 1. The extracted ion chromatograms were integrated, and peak areas were used to calculate the total protein quantity of each individual sample.

### Validation assays

In order to corroborate some of the findings of the proteomic analysis, three assays were performed in plasma samples from all sampled fish, including the ones used in the proteomics study.

#### Plasma complement assay

The lytic activity of the alternative complement pathway (ACP) was determined using sheep red blood cells (RBCs; Thermo Fisher Scientific, Waltham, MA, USA) as targets, and the dilution corresponding to 50% haemolysis ml^−1^ was expressed as alternative complement activity (ACH_50_). This assay was performed following the procedure described in [33], in sample duplicates using 2.85 × 10^8^ sheep RBCs ml^−1^.

#### Plasma cholesterol assay

Plasma cholesterol was measured using the Infinity Cholesterol Liquid Stable Reagent (Thermo Fisher Scientific), following the manufacturer’s instructions. A calibration curve was performed using serial dilutions of Cholesterol (0–780 mg dl^−1^; Sigma-Aldrich, St. Louis, MO, USA). The amount of plasma per reaction was 4 µl. Reactions were performed in duplicate.

#### Plasma biotin detection

Plasma biotin was measured using the Biotin Quantitation Kit (Abcam, Cambridge, UK) following the manufacturer’s instructions, using 30 µl of plasma, in duplicate. Biotinylated bovine serum albumin was used as a positive control. A standard curve was prepared using biotin concentrations ranging between 20 and 1000 µM.

### Data and statistical analysis

The protein areas obtained with PeakView® v2.1 software (AB Sciex) were normalised by the total sum of the areas of all the quantified proteins. Normalised data were used to build a partial least squares-discriminant analysis (PLS-DA) model using the Bioconductor R package ‘ropls’ [34]. The quality of th ePLS-DA model was evaluated with the fit [R2Y(cum)] and prediction [Q2 (cum)] indicators. A validation test consisting of 500 random permutations was performed to discard overfitting of the PLS-DA model. The contribution of the different proteins to the group separation was determined by variable importance in projection (VIP) values. A VIP value > 1 was considered to be the threshold to determine discriminant proteins in the PLS-DA model [35–37]. Hierarchical clustering, heatmap representation and K-means analyses were performed with the normalised area values of all discriminant proteins (VIP > 1) using iDEP.95 web application [38].

To perform a pathway analysis, the discriminant protein identifiers were converted to their human equivalents, when possible, and analysed with the Bioconductor ‘ReactomePA’ R package [39].

The R package *corrplot* was used to calculate correlations between the different proteins and the infection intensity applying the *cor.test* function to compute significant correlation coefficients with a confidence level of 0.95.

All data were checked for normality prior to any statistical analysis. Statistical differences between C and R (L, M, H) Hb, ACP, cholesterol and biotin values were calculated using a one-way analysis of variance and a post hoc multiple comparisons Holm-Šídák test. Differences were considered significant at *P* < 0.05, and a power analysis was performed in every test. All statistical analyses were performed using SigmaPlot v.14.0 (Systat Software, Inc., San Jose, CA USA).

## Results

### Plasma proteome analysis

**Fig. 1:**
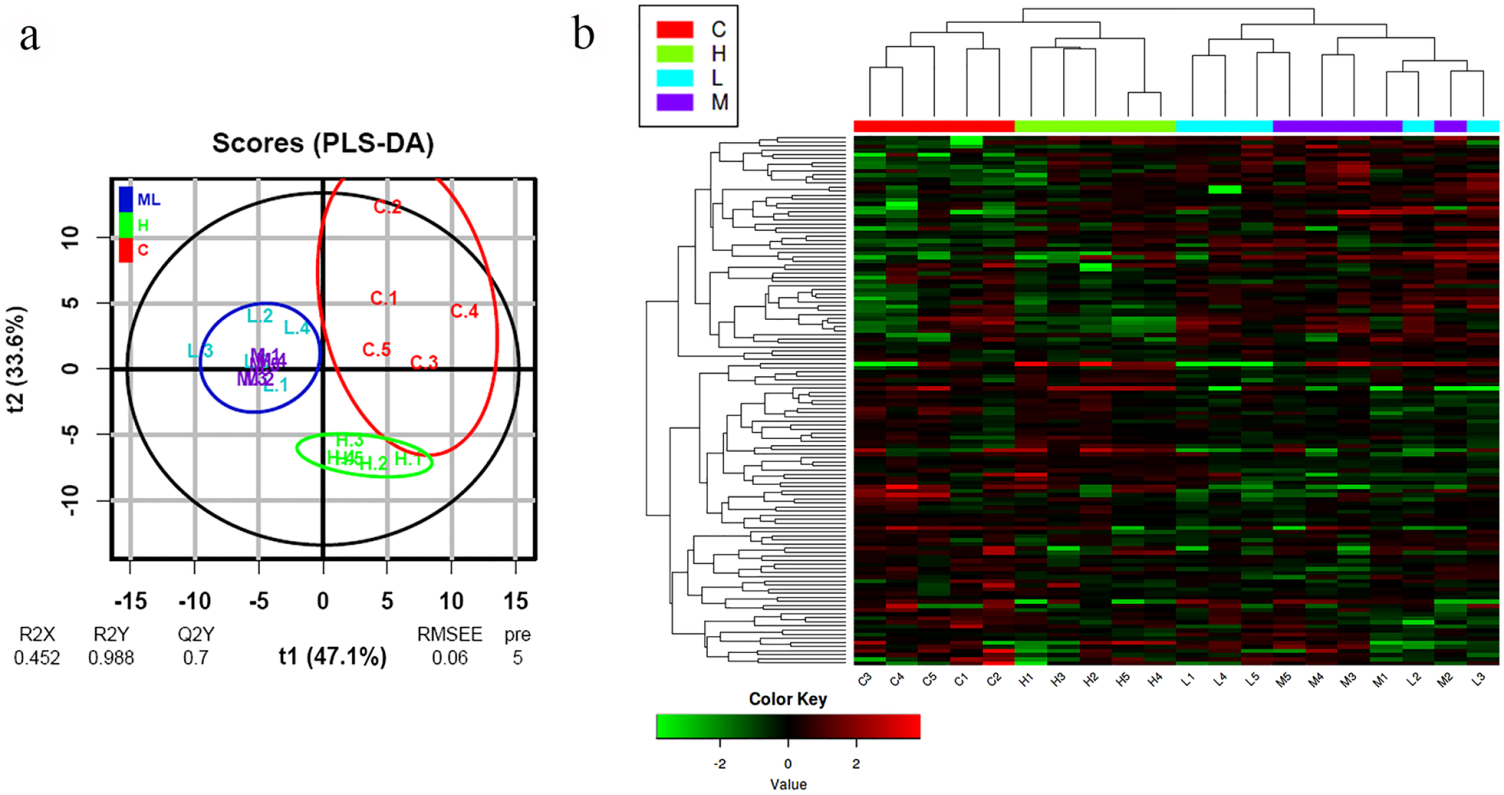
**a** Two-dimensional PLS-DA score plot representing the distribution of the samples between the first two components of the model (t1 & t2). Uninfected fish [Control (C); *n* = 5] are represented in red; *Sparicotyle chrysophrii*-infected fish are represented in different colours according to level of infection (worms fish^—1^), with green indicating a high level of infection (H; *n *= 5), violet indicating a medium level of infection (M; *n* = 5) and blue indicating a low level of infection (L; *n* = 5). Ellipses represent the Mahalanobis distance. R2X and R2Y represent the fraction of the variance of the* X* and* Y* matrix, respectively (explained variance). Q2Y represents the predictive accuracy of the model. Values that approximate 1 indicate an effective model. RMSEE represents the square root of the mean error between the actual and the predicted responses. The model was constructed using five components (pre = 5). **b** Heatmap representing the abundance distribution (*Z*-score) of the 129 proteins identified to be driving the separation among groups in **a**). Dendrograms represent hierarchical clustering of proteins (rows) and samples (columns). Samples are colour coded following the same criteria as in **a**. PLS-DA, Partial least squares-discriminant analysis

A total of 291 GSB proteins were identified and quantified in the whole set of plasma samples. The normalised abundance values of each individual sample were used to construct a PLS-DA model to determine differences amongst groups. The PLS-DA model was based on five components (Additional file 2: Figure S2) which explained 98.8% (R2Y) and predicted 70% (Q2Y) of the total variance (Fig. 1a). The model was validated using a permutation test (Additional file 2: Figure S2), and no outliers were detected during this analysis (Additional file 2: Figure S2). The PLS-DA model clearly separated the C group from fish in the R group. The dispersion of the C samples in the plot showed great individual variability in this group (Fig. 1a). Highly infected fish (H) formed a separate group, whereas fish with medium and low levels of infection (M/L) were not significantly separated by the model and constituted a single set. Recipient fish (H and M/L groups) showed lower variability in their proteomic profiles than the C group. A PLS-DA model using the sampling point as variable, instead of infection intensity, was constructed to determine whether differences could be due to the sampling time. As this model failed, we proceeded with the results showing differences by infection intensity.

**Fig. 2:**
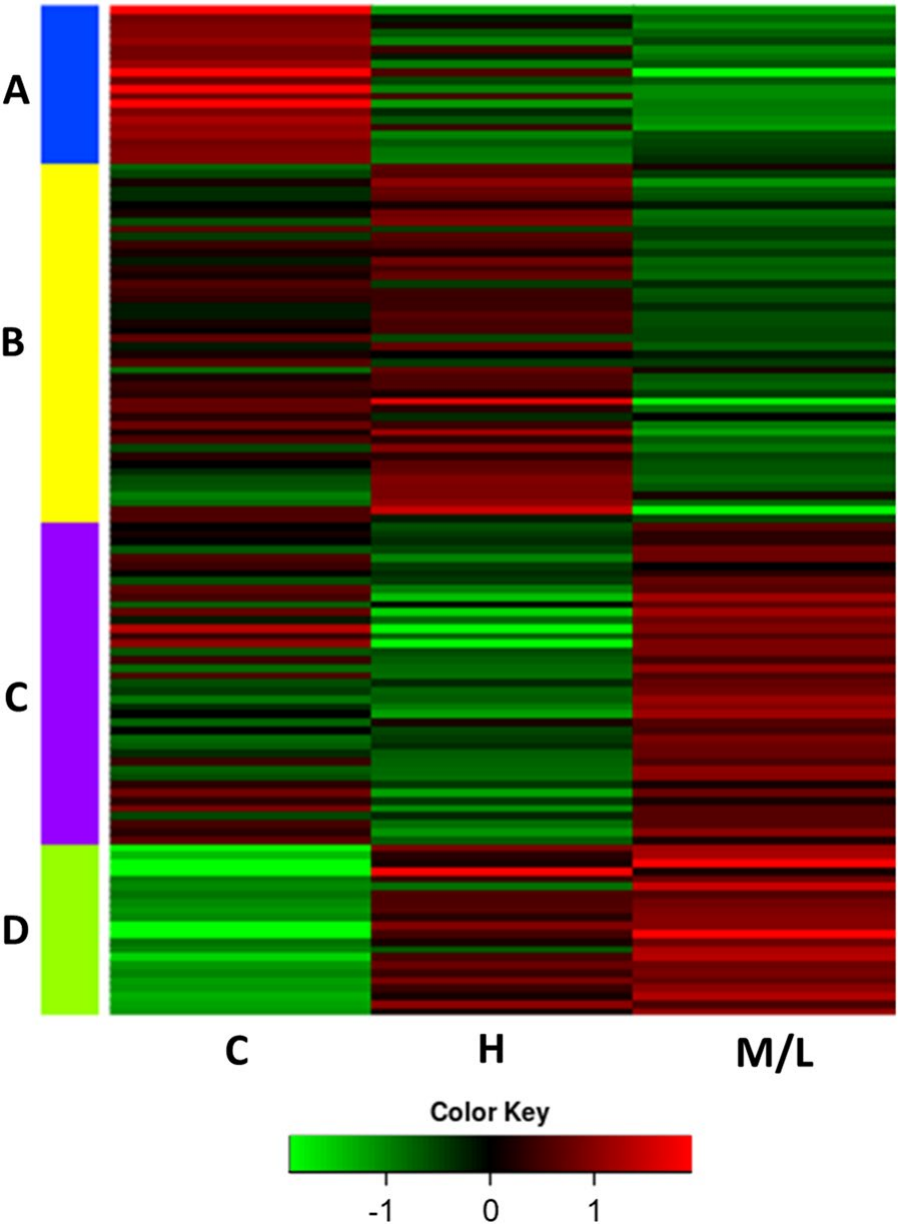
K-means analysis separating the 129 discriminant proteins into four clusters based on expression level in the different groups (*Z*-score). Different colours indicate different clusters, with blue indicating cluster A (*n* = 20), yellow indicating cluster B (*n* = 46), violet indicating cluster C (*n* = 41) and green indicating cluster D (*n* = 22). Group means are represented for clarity. C, control (uninfected) fish; H, fish with high degree of infection; M/L, fish with medium/low degree of infection

The PLS-DA model yielded 129 proteins with VIP values > 1 which are responsible for the separation of the proteomic profiles among the three groups (Additional file 3: Table S1). These differentially abundant proteins driving the separation of the different groups were further explored in a heatmap. Hierarchical clustering once again showed a clear separation into three groups: C, M/L and H, validating the results obtained from the PLS-DA (Fig. 1b). K-means analysis, conducted to visualise patterns among the differentially abundant proteins, revealed four clear clusters (Fig. 2). Cluster A consisted of 20 proteins that were more abundant in the C samples than in R samples, among which immunoglobulin proteins prevailed. Cluster B grouped 46 proteins that were more abundant in the highly infected samples (H) than in the other two groups. Cluster C contained 41 proteins with a high abundance in the M/L group, a low abundance in the H group and intermediate values in the C group. Cluster D comprised 22 proteins with a low presence in C samples and increased presence in R fish.

### Pathway analysis

**Fig. 3:**
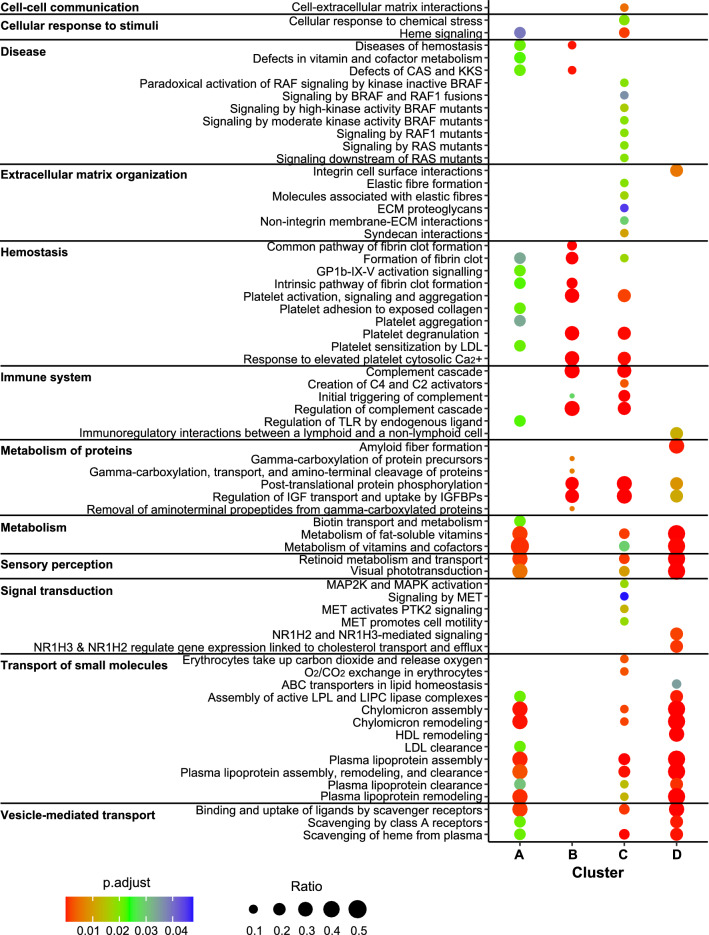
Dot plot pathway enrichment map showing significantly overrepresented pathways (left) (*P*adj. < 0.05) in the lists of proteins obtained for the different clusters in the K-means analysis (right) represented in Fig. 2. The colour of the dots represents the *P*adj. value, and the size of the dots represents the proportion of proteins relative to the total amount of proteins for each pathway

In an attempt to clarify the biological significance of the changes observed in the plasma proteome of the different groups, pathway analysis was performed with the differentially abundant proteins classified in the four K-means clusters. Enriched pathways (*P*adj. < 0.05) in R GSB were coherent with functions expected to be found in plasma, highlighting an enrichment in pathways related to haemostasis, immune system, metabolism of vitamins and proteins, and transport of lipoproteins or O_2_/CO_2_ (Fig. 3). Among the pathways associated with the immune system, the complement system was highly represented. Overall, the most represented function was related to lipid (cholesterol) transport. Only 15 of the 129 proteins (11.63%) could not be converted into human equivalents and were not considered for the pathway analysis.

### Proteins correlated with infection intensity

Correlation analysis revealed that 22 proteins were significantly correlated with the infection degree (Table 2). Fourteen proteins, including three apolipoproteins, two globins, complement component 3 (C3), ceruloplasmin and biotinidase, were negatively correlated with the infection intensity. Conversely, eight proteins exhibited a positive correlation with the disease.

**Table 2:** Plasma proteins whose abundance was significantly correlated with *Sparicotyle chrysophrii* infection degree

Protein	Correlation coefficient^a^	*P*-value^b^
Saxitoxin and tetrodotoxin-binding protein 1-like	− 0.65	0.002
Alpha-2 globin	− 0.62	0.003
Immunoglobulin light chain, partial	− 0.61	0.005
Biotinidase	− 0.58	0.007
Apolipoprotein B-100	− 0.56	0.010
Ectonucleotide pyrophosphatase/phosphodiesterase family member 2	− 0.54	0.014
Complement C3-like	− 0.51	0.020
Apolipoprotein H	− 0.50	0.024
Carboxypeptidase N subunit 2	− 0.49	0.030
Beta globin	− 0.48	0.033
Apolipoprotein A-II	− 0.48	0.033
Ceruloplasmin	− 0.47	0.037
Serpin family G member 1	− 0.45	0.048
Ladderlectin-like	− 0.45	0.048
Immunoglobulin lambda chain C region	0.57	0.009
Hibernation-specific plasma protein HP-55	0.56	0.010
L-rhamnose-binding lectin CSL2	0.53	0.016
Alpha-*1*-microglobulin	0.52	0.018
Cyclin dependent kinase like 1	0.47	0.036
Serotransferrin	0.46	0.039
Coagulation factor X-like	0.46	0.040
Estrogen-regulated protein	0.45	0.048

^a^Negative and positive correlations and the strength of each correlation are shown by the sign and value of the correlation coefficient

^b^Significant correlations were assumed at *P-*value < 0.05

### Validation assays

**Fig. 4:**
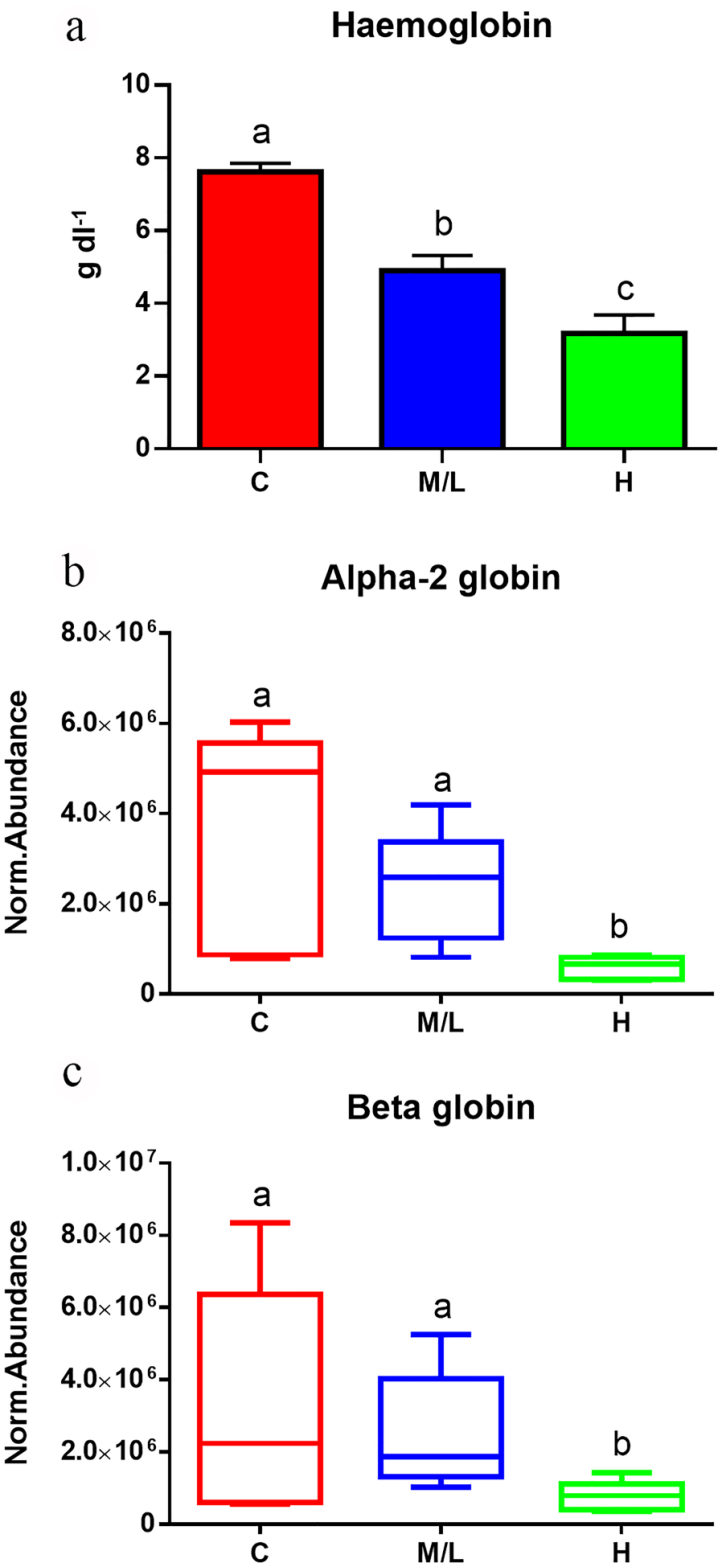
**a** Haemoglobin values measured in control fish (C, *n* = 50) and in *S. chrysophrii*-infected fish with a medium/low (M/L, *n* = 31) and high (H, *n* = 16) infection degree.** b**,** c** Normalised protein abundance values of alpha-2 globin (**b**) and beta-globin (**c**) measured by proteomics in plasma samples of C fish (*n* = 5) and in M/L (*n* = 10) and H (*n* = 5) infection groups. Values are presented as the mean ± SEM (standard error of the mean). Statistical differences among groups at *P* < 0.05 are noted with different letters (Kruskall-Wallis test)

**Fig. 5:**
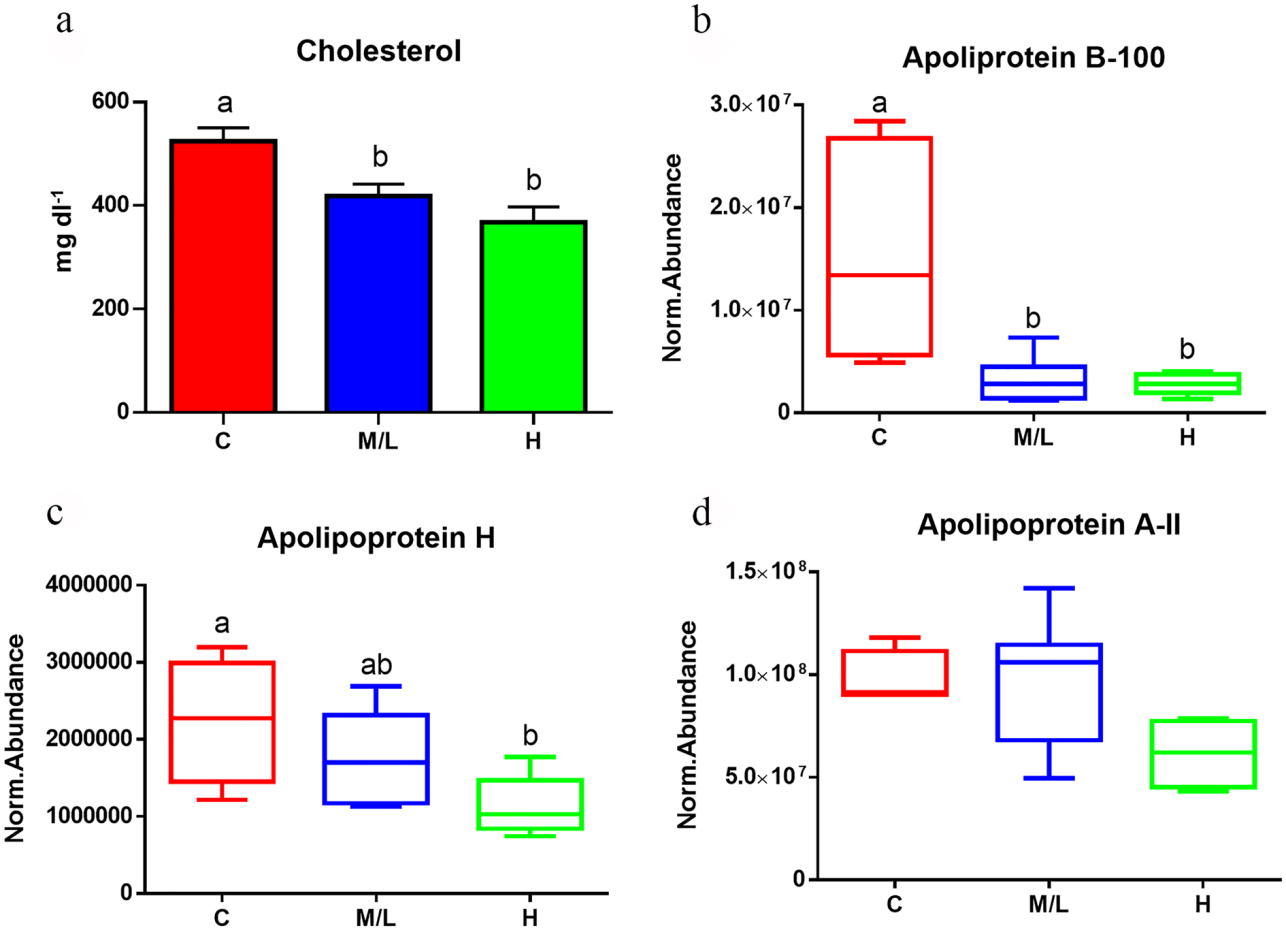
**a** Plasma cholesterol values measured in control fish (*n* = 50) and in *S. chrysophrii*-infected fish with a medium/low (M/L, *n* = 31) and high (H, *n* = 16) infection degree.** b**–**d** Normalised protein abundance values of apolipoprotein B-100 (**b**), apolipoprotein H (**c**) and apolipoprotein A-II (**d**) measured by proteomics in plasma samples of C fish (*n* = 5) and in the M/L (*n* = 10) and H (*n* = 5) infection groups. Values are presented as the mean ± SEM. Statistical differences among groups at *P* < 0.05 are noted with different letters [one-way analysis of variance (**a**, **c**, **d**) or Kruskall-Wallis test (**b**)]

**Fig. 6:**
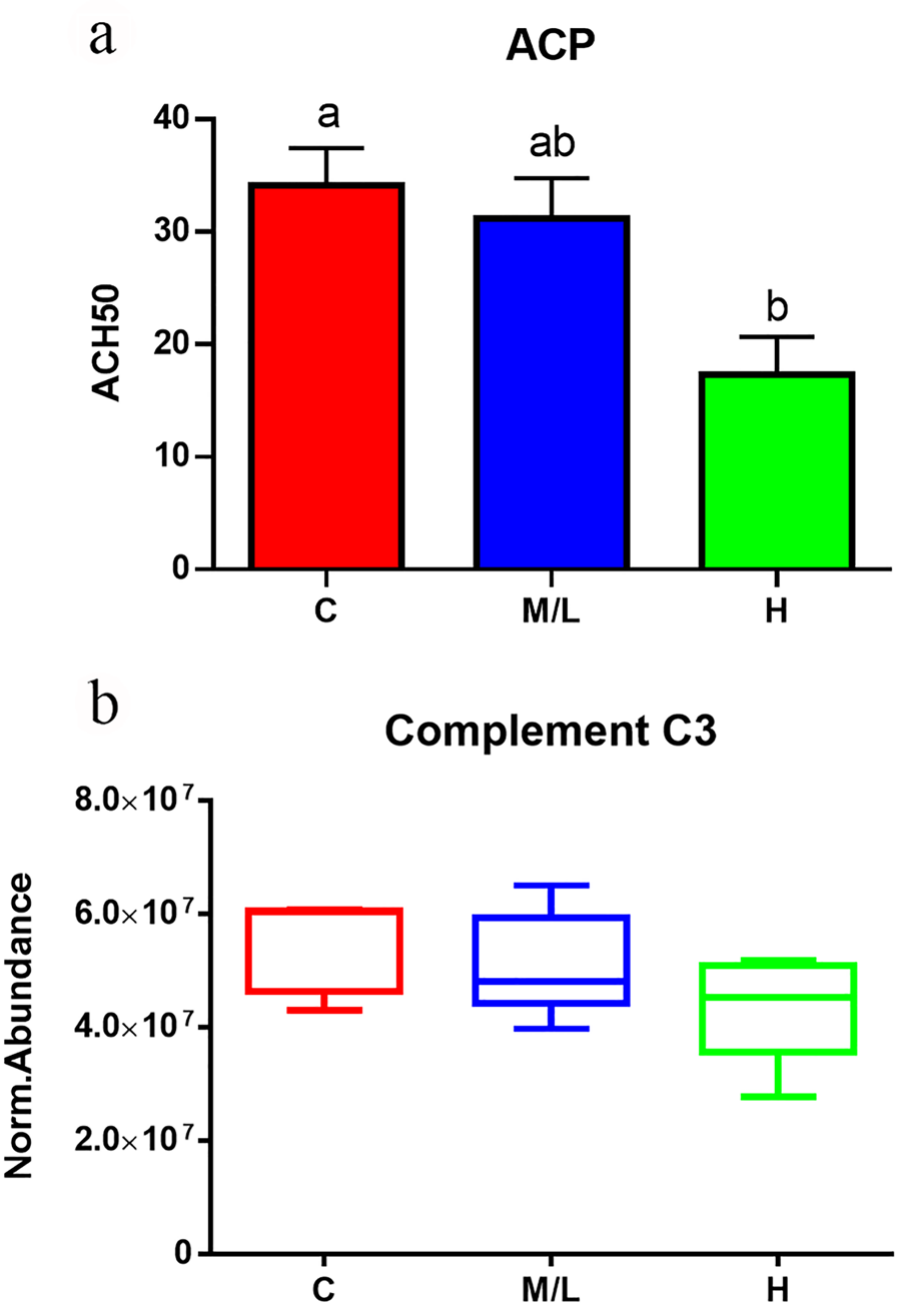
**a** Activity of plasma alternative complement pathway (ACP) measured in control fish (*n* = 50) and in *S. chrysophrii*-infected fish with a medium/low (M/L, *n* = 31) and high (H, *n* = 16) infection degree. **b** Normalised protein abundance values of complement component 3 (C3) protein measured by proteomics in plasma samples of C fish (*n* = 5) and in the M/L (*n* = 10) and H (*n* = 5) infection groups. Values are presented as the mean ± SEM. Statistical differences among groups at *P* < 0.05 are noted with different letters (one-way analysis of variance)

Haemoglobin values (Fig. 4), plasma cholesterol concentrations (Fig. 5) and ACP (Fig. 6) showed a gradual and significant decrease with increasing infection intensity. These results validated the detected gradual decline in plasma alpha-2 and beta globins, apolipoproteins B-100, H and A-II and C3 at the protein level. On the other hand, no significant differences were detected in plasma biotin levels, and the measured values were very close to the lower detection threshold of the technique (Additional file 4: Figure S3). It is to be noted that biotinidase levels were also in the low range, preventing us from drawing firm conclusions about the detected differences.

## Discussion

Despite the impact of *S. chrysophrii* on infection on animal welfare and the economic repercussions, little is known about the effect of *S. chrysophrii* in GSB. The results of the present study on plasma proteomics of *S. chrysophrii*-infected GSB provide information that will enable a better understanding of the pathogenesis of sparicotylosis, providing evidence on the metabolic pathways affected by the disease under different infection intensities. Furthermore, these results may shed some light upon which host resources are required by this monogenean species to ensure its survival as a first approach to elucidate the unknown pathogenicity mechanisms of the parasite and ultimately suggest immune-evasion strategies.

The discriminant analysis of the abundance of all detected proteins formed three different groups (C, M/L and H). The dispersal of these proteins among the healthy GSB (C) group was clearly greater than that among diseased fish (M/L and H groups) (Fig. 1a). However, differentially abundant proteins in the M/L group showed a greater disparity than those in the H group, which in turn seemed closer to the C group phenotype in both the PLS-DA model and heatmap (Fig. 1). Large variability is always an expected feature in control samples; in our study, the fish were obtained from a commercial farm and were not clonal lines or even from a single family. Although they came from the same production batch, they probably had very different genetic backgrounds, which would provide variable results. The observed decrease in variability during infection is interesting, as it points towards a homogeneous response elicited by/against the parasite, with all fish responding with the same trend. These observations suggest that the hosts suffer a profound imbalance with mild (M/L) *S. chrysophrii*-infection intensities, as observed by Piazzon et al. [19]. In GSB that survived to high parasitic burdens (H), the plasma proteomic profile closely resembled that of C fish, possibly reflecting the onset of compensation mechanisms to restore homeostasis, such as haemostatic events. During the progress of sparicotylosis under experimental conditions, this apparent recovery of the plasma’s proteomic profile might be the result of the monogeneans’ intimate coevolution with their hosts. Monogenean evolution is associated with strict host specificity and coevolution between worm and host [40, 41], which, in light of the results of the present study, might include attenuation of the worm’s pathogenicity in order to assure the host’s survival and parasite’s persistence. Under farming conditions, however, which include high stocking densities, exposure to environmental stressors and multiple pathogen offenders, high infection pressures and recurrent sparicotylosis infections once the disease is established, high mortality is reported, even after the fish has received treatment. Apparently, under these harsh farming conditions, fish would be unable to recover homeostasis, resulting in the combined pathogenic effect of the parasite with the biotic and abiotic stressors, including secondary opportunistic infections, being more devastating. Hence, future proteomic plasma studies of moribund GSB from sea cages suffering sparicotylosis would shed some light on this issue. However, the current results were obtained under experimental conditions, in a time-limited exposure (10 weeks) to a single purified pathogen offender, in contrast to an enzootic farm. The potential therapeutic importance of such a natural attempt of homeostatic restitution in highly infected fish under experimental conditions is paramount, since a strategy based on an earlier stimulation of these mechanisms by dietary or health interventions would open the path to mitigation of the effects of the disease. *Sparicotyle chrysophrii*-modulated proteins are involved in several biological processes in GSB. Among these, the levels of various proteins increased while those of others decreased in a complex network of interactions. The main pathways severely modulated by *S. chrysophrii* were those related to haemostasis, lipid metabolism and transport and the immune system (Fig. 3).

### Haemostasis

Polyopisthocotylean monogeneans have been described as haematophagous parasites [42, 43], but it has not been until recently that the haematophagous nature of *S. chrysophrii* has been experimentally demonstrated [44]. From the current study, we can discern a clear negative impact on haemostasis in GSB. Blood Hb levels significantly dropped as the parasite burden increased (Fig. 4). These low Hb values mirrored the plasma proteomic results, which showed that the main Hb constituents, alpha-2 and beta globins, negatively and significantly correlated with the infection intensity (Table 2; Fig. 4). In addition, alpha-1-microglobulin, a radical scavenger associated with haem toxicity and erythroprotective anti-haemolytic effects in humans [45], presented a positive and significant correlation (Table 2). This suggests that *S. chrysophrii*-infected GSB suffer from haemolytic anaemia, as an increase in alpha-1-microglobulin occurs in hosts facing a haemolytic insult, which leads to the release of Hb and free haem groups from erythrocytes, increasing the oxidative stress [45]. Overall, these results would imply anaemia and oxygen transport impairment, explaining the hypoxia and lethargy signs observed in parasitised fish. Furthermore, the specific mechanisms this parasite uses for blood-feeding are still unknown, but our preliminary results on the matter suggest that *S. chrysophrii* is able to feed on blood meals resulting from GSB RBC lysate. The exact mechanism remains to be elucidated.

The coagulation cascade also seems to be triggered by sparicotylosis. In mammalian [46, 47] and fish [48] blood, the extrinsic pathway is initiated following tissue damage and subsequent exposure of subendothelial tissue factor (TF) to coagulation factor VII, whereas the intrinsic pathway is triggered by the exposure of a foreign negatively charged surface to coagulation factor XII. Both pathways converge in coagulation factor X, after which the common pathway of the coagulation cascade follows, resulting in the production of thrombin and leading to clot formation and final restoration of haemostasis [46, 47]. In the current study, most proteins involved in the coagulation cascade were represented in cluster B of the K-means analysis (Fig. 2). In GSB suffering sparicotylosis, both intrinsic (coagulation factor IX) and extrinsic (factor VII) pathways of the coagulation cascade as well as the common pathway of the coagulation cascade (factors X and V) were modulated (Additional file 3: Table S1). It is noteworthy that all coagulation factors in cluster B were upregulated in the H group, but not in the M/L group (Fig. 2), which is in agreement with the significantly positive correlation of factor X with the infection intensity (Table 2). Thus, the coagulation capacity of GSB apparently increased when high parasitic burdens were reached. Similarly, in several tick species, different proteins with anticoagulant properties affecting the intrinsic, extrinsic and common coagulation pathways [49–51] have been described and characterised, suggesting that these haematophagous parasites can modulate their host’s haemostasis at different levels.

Since *S. chrysophrii* is an ectoparasite and not an intravascular parasite, we suspect that the intrinsic pathway could be, in part, triggered by remnants of RBCs [52] resulting from haemolysis. In contrast, the activation of the extrinsic pathway may be due to tissue disruption induced by the parasite’s haptor and feeding mechanisms.

### Lipid metabolism and transport

Different parasitic species, ranging from Protozoa to Metazoa, have been described as being able to alter the lipidic profile of their host species, both in fish [53] as well as in higher vertebrates [54–63]. In particular, Platyhelminthes are unable to synthesise fatty acids de novo [64], thus relying on the host’s lipid reservoir to ensure their survival. Several fatty acid-binding proteins (FABPs) have been identified in trematode species, such as *Schistosoma* spp., *Fasciola* spp and, most recently, in the diplozoid monogenean *Eudiplozoon nipponicum* [65, 66]. Although FABPs have been described to play a role in fatty acid uptake by *Fasciola hepatica* from host blood and in immunomodulation, their function in monogeneans remains unknown [65]. Our results show that apolipoprotein B-100 (ApoB-100) and apolipoprotein A-II (ApoA-II) levels were negatively and significantly correlated with the infection intensity (Table 2; Fig. 5). In agreement with this observation, the expression of apolipoproteins was found downregulated in the liver and spleen of GSB with a mild *S. chrysophrii* infection [19]. In addition, cholesterolaemia values in plasma samples of *S. chrysophrii*—infected GSB were significantly lower than those in the C group, supporting our proteomic results (Fig. 5). The reduction in plasma cholesterol levels in GSB was also triggered by environmental stressors [67, 68]) and dietary intervention involving the replacement of fish meal and oil by vegetable ingredients [33, 69]. The latter provoked a simultaneous drop in plasma cholesterol and blood Hb, which was reversed by a butyrate additive in the diet. This finding could open a path for the use of butyrate as a mitigation strategy for the effects of sparicotylosis.

ApoB-100 is a crucial structural component in very-low-density lipoproteins (VLDL) and low-density lipoproteins (LDL), which are predominantly composed of triglycerides and cholesteryl esters, respectively. ApoA-II, on the other hand, is associated with high-density lipoproteins (HDL_2_ and HDL_3_) that are predominantly composed of cholesteryl esters [70, 71]. Our results suggest a dependency of *S. chrysophrii* on its host’s lipid reservoir, but the function of these lipids is intriguing. In the case of the previously mentioned trematode species *Schistosoma* spp. and *Fasciola* spp. and the diplozoid monogenean *E. nipponicum*, it has been suggested that the host’s incorporated lipids may play important roles in maintaining different cellular structures after their distribution and storage in the parasite’s body or they may also be found in excretion/secretion products, which are involved in the modulation of the host’s immune system [66, 67]. The human trematode *Schistosoma mansoni* has been described to bind part of the host’s LDL to their surface, which might be an immune evasion strategy, in addition to ingesting LDL, breaking it down and distributing lipids throughout the worm [72]. *Sparicotyle chrysophrii* might be using similar strategies since it seems to preferentially rely on the easier digestible and transportable smaller sized VLDLs and LDLs of the host, but there is still no evidence for the worm feeding or displacing GSB’s lipoproteins.

Plasma lipoproteins (LDLs and HDLs) play an essential role in host defence as a component of the immune system [73–75] and against bacterial, viral and parasitological infections [73] in mammals. Hence, in our fish-parasite model, an alteration in lipoprotein levels could render the host more susceptible to secondary infections. Moreover, other roles in haemostasis have been granted to LDLs and HDLs, thus somewhat contributing to the control of haematological parameters, such as RBC membrane stability [76].

### Immune system

Differences in the host’s immune response have been observed between monopistholocotylean and polyopisthocotylean monogenean parasites [77]. These differences have been suggested to lie in the different feeding strategies [77] since polyopisthocotylean monogeneans are generally considered to be haematophagous and, therefore, need to evade the host’s immune response to ensure their feeding and survival. Our study shows how *S. chrysophrii* infection changes the abundance of several complement proteins (factor H, factor B, factor I, C1q, C3, C4, C5, C6, C7, C8; Additional file 3: Table S1), inducing an inhibition of the alternative pathway as the infection intensity increases (Fig. 6). Along the same line, other studies with the same host and parasite species have revealed that this parasite downregulated *c3* splenic expression, upregulated complement factor H (complement inhibitor) in spleen and gills [19] and lowered complement levels in serum [18]. A local downregulation of *c3* expression has also been described in other monogenean infections [78, 79]. We observed that the depletion of complement effectors in GSB plasma worsened during the infection, compromising the fish immunocompetence. It is also noteworthy that the presence of immunoglobulin chains, mostly variable regions of the light chains, among the proteins were significantly less abundant in R fish, regardless of their infection intensity (cluster A). This result indicates that B cells are being modulated upon parasite infection, with a probable shift of the immunoglobulin (Ig) repertoire. Modulation of immunoglobulin transcripts, including light chains and variable genes, have also found at a transcriptomic level in gills (local) and spleen (systemic) in mild *S. chrysophrii* infections [19]. Further studies elucidating Ig titres and the Ig repertoire at systemic and local levels are needed to determine if this is due to an inhibition of antibody production or a shift towards a focused and specific response. Our results point to a complex network regulating the innate immune response, including SERPINs and ceruloplasmin, which may indirectly modulate the complement system, resulting in neutrophil activation and inflammation.

### Proteins linking haemostasis and the immune system

#### Serine-protease inhibitors

Serine proteases are enzymes that have been highly conserved during evolution which play crucial roles in several physiological processes, including blood coagulation, fibrinolysis, inflammation and immune response. Serine-protease inhibitors (SERPINs) obtain their name from serine protease inhibitors. They are a superfamily of proteins that primarily regulate the proteolytic pathways of serine and cysteine proteases [80–83]. It has been described that protease inhibitors may have a leading role in host-parasite interactions and, more specifically, in evasion mechanisms and survival on the parasite’s behalf [84–86].

In the current study, three SERPINs were differentially expressed following infection; SERPINA1 (α_1_–antitrypsin), SERPIND1 (heparin coagulation factor II) and SERPING1 (C1-inhibitor; C1INH) (Additional file 3: Table S1). SERPINA1 was grouped in cluster C (more abundant in the M/L group), whereas both SERPIND1 and SERPING1 were grouped in cluster B (more abundant in the H group) (Fig. 2).

SERPINA1 inhibits neutrophil elastase, a serine protease with microbiocidal effects that is involved in the acute phase of the inflammation process and tissue remodelling [87]. We relate this observation to an early impairment of an inflammatory response elicited by neutrophils. This event could be driven by either a GSB response towards a traumatic event involving tissue disruption or by deliberate modulation of SERPINA1 by *S. chrysophrii* as an evasion mechanism, with the ultimate aim to enable attachment to gill filaments. It has recently been suggested that some digenean trematode parasites could have the ability to modulate the host’s SERPINs [25], but no published data are are available on SERPINA family members being modulated by fish parasites.

Both SERPIND1 and SERPING1 play key roles in coagulation; however, SERPING1 also affects the immune system. SERPIND1 is known to, directly and indirectly, inhibit thrombin in the common pathway of the coagulation cascade [80, 81, 88], thus preventing fibrinogen and platelet activation and ultimately preventing clot formation and haemostasis restoration. At the haemostatic level, SERPING1 inhibits several components within the intrinsic coagulation pathway [plasma kallikrein, activated factors XII (FXIIa) and XI (FXIa)] as well as fibrinolytic proteases (plasmin, tPA and uPA). Further SERPING1 inhibitory abilities extend to both C1s and C1r, modular proteases responsible for the activation and proteolytic activity of the C1 complex of the classic complement pathway [46, 47, 80, 81, 83, 89–91].

Thus, the significantly higher abundance of SERNPIND1 and SERPING1 in GSB with high parasitic burdens could imply an anticoagulant and innate immunosuppressor effect in these hosts (Fig. 2). However, within cluster B, SERPING1 shows a significant negative correlation with the infection intensity (Table 2). SERPINs operate within a complex physiological modulation network, and further SERPIN-targeted studies are needed to unravel this paradox, as well as the opposing coagulant and anticoagulant actions of serpins and the coagulation cascade. Counter-regulation evidenced by our results might be the effect of the host response aiming for homeostatic balance or a host versus parasite modulation.

#### Ceruloplasmin

Ceruloplasmin is an acute-phase protein that has been associated with inflammation, severe infection and tissue damage in mammals and fish. Ceruloplasmin has also been described as a copper-carrying protein, ultimately playing a role in hypoxic vasodilation and ischaemia–reperfusion cytoprotection [92–94] and having the ability to oxidise toxic plasma ferrous iron into its ferric form to be transported by transferrin [93]. Moreover, under hyperammonaemia conditions, the intrinsic pathway of the coagulation cascade is triggered, and the functional activity of platelets decreases. However, ceruloplasmin can prevent haemostatic disorders by restoring platelet functionality and preventing hypercoagulation [95].

Henry et al. [18] previously described no significant differences in ceruloplasmin activity in GSB after a 10 week-long *S. chrysophrii* infection. Our results suggest an initial increase in plasma ceruloplasmin levels in M/L fish followed by a later decrease during the course of infection in the H group. Thus, we hypothesise that fish with lower infection intensities were in an acute phase of the disease, while H GSB restored their ceruloplasmin to control levels later during the progress of infection. However, comparison of our results with previously published findings [18] is however complicated since experimental designs and the infection outcome were very different.

## Conclusions

Understanding how GSB responds to *S. chrysophrii* is critical for developing new treatments and health management strategies in the aquaculture industry. The present plasma proteomic study of *S. chrysophrii*-infected GSB provides a crucial global overview of the pathogenesis of sparicotylosis, representing a valuable contribution to the understanding of the disease and highlighting new targets for further research. However, our results are based on the disease progression of a pure *S. chrysophrii* experimental infection, which does not totally depict farming conditions. Sparicotylosis profoundly alters the haemostasis, the innate immune system and the lipid metabolism and transport in GSB. However, in high-intensity experimental infections, GSB seems to attempt to restore some of the alterations suffered during the acute phase of the disease. This could be either due to the close evolutionary ties between *S. chrysophrii* and GSB, or to a host protection mechanism against the damage caused by the activation of acute mechanisms.

## Supplementary Information

**Additional file 1: Figure S1. **Schematic representation of samples taken in each sampling and their downstream use.

**Additional file 2: Figure S2. **Partial least-squares discriminant analysis model overview depicting the optimal number of components used to build the model (p1-p5). The* Y*-axis represents the cumulative fit (R2Y) and prediction (Q2Y) coefficients for each of the components (**A**). Validation of the model (permutation test, 500 permutations) to estimate R2Y and Q2Y significance. pR2Y and pQ2 are considered significant at *P* < 0.05 (**B**). Observation diagnostics was performed to detect outliers by plotting the score and orthogonal distances of each sample (red = control fish; blue = fish with low/medium infection degree; green = fish with high infection degree). No outliers were detected in this model (C).

**Additional file 3: Table S1. **List of proteins with VIP value > 1 responsible for the separation of groups in the PLS-DA model presented in Fig. 1. ordered by the corresponding cluster from the K-means analysis (Fig. 2). The group mean normalised abundance values are shown on the right.

**Additional file 4: Figure S4. **Plasma biotin values measured in control (C, *n* = 50) and *Sparicotyle chrysophrii*-infected fish with a medium/low (M/L, *n* = 31) and high (H, *n* = 16) infection degree (**A**). Normalised protein abundance values of biotinidase (B) measured by proteomics in plasma samples of control (C, *n* = 5), medium/low (M/L, *n* = 10), and high (H, *n* = 5) infection groups. Values are represented as mean ± SEM and statistical differences among groups are noted with different letters (Kruskall-Wallis test, *P* < 0.05).

## Data Availability

The proteomics data has been deposited in the PRIDE repository, with the dataset identifier PXD034541. The rest of the data generated by this study is included in the manuscript and in the additional files.

## Abbreviations

ACP: Alternative complement pathway
C: Control
GSB: Gilthead sea bream
H: High level of infection
L: Low level of infection
M: Medium level of infection
R: Recipient
SWATH-MS: Sequential window acquisition of all theoretical mass spectra
