# Supplementary figures and images for: A bloody interaction: plasma proteomics reveals gilthead sea bream (Sparus aurata) impairment caused by Sparicotyle chrysophrii
Source: Parasit Vectors. 2022 Sep 10;15:322. doi: 10.1186/s13071-022-05441-1 (PMC9463799; doi:10.1186/s13071-022-05441-1)

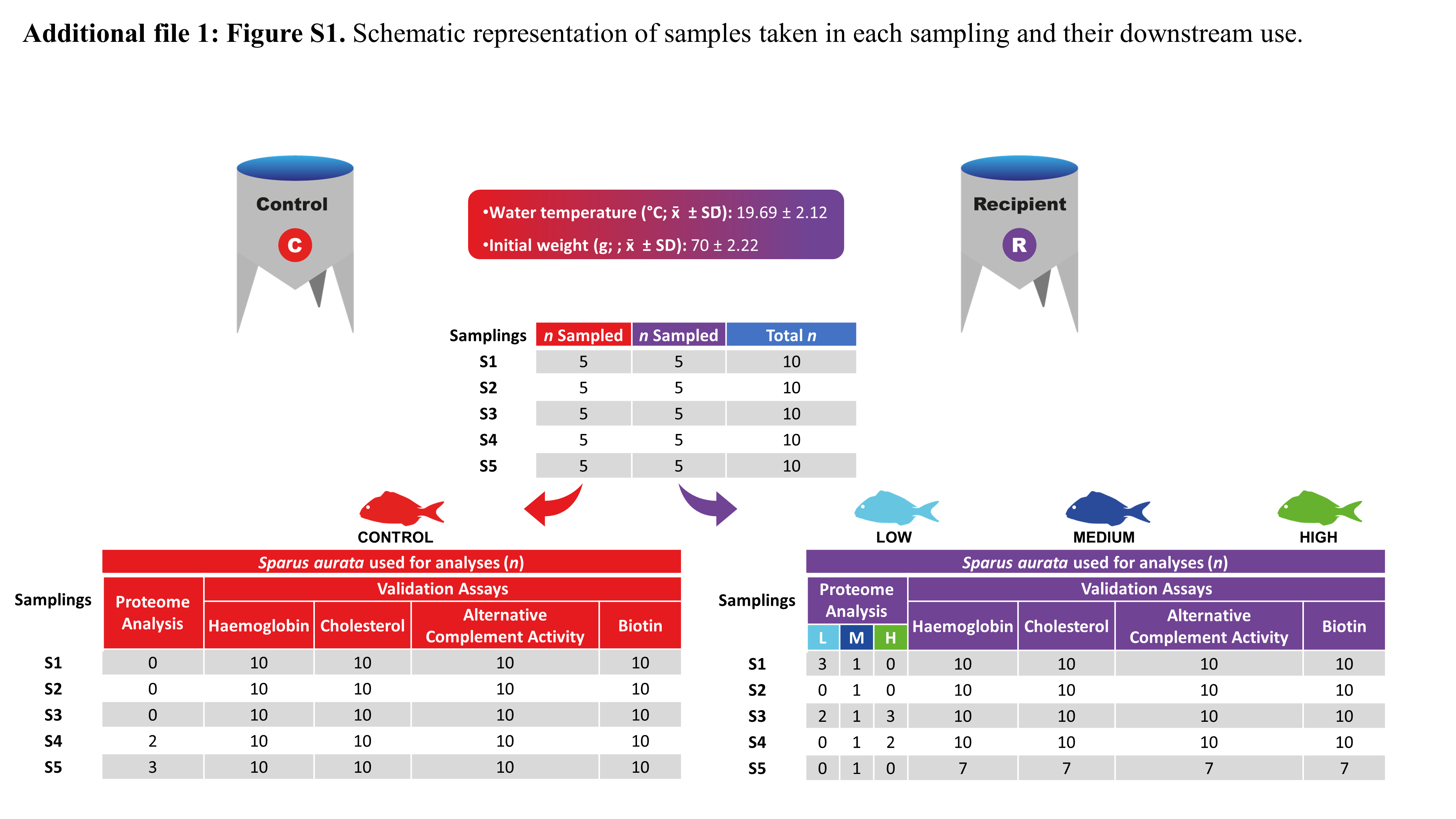

Supplement: Supplementary file 1 — Additional file 1: Figure S1. Schematic representation of samples taken in each sampling and their downstream use. [file 13071_2022_5441_MOESM1_ESM.tif]
