# Supplementary material for: A bloody interaction: plasma proteomics reveals gilthead sea bream (Sparus aurata) impairment caused by Sparicotyle chrysophrii
Source: Parasit Vectors. 2022 Sep 10;15:322. doi: 10.1186/s13071-022-05441-1 (PMC9463799; doi:10.1186/s13071-022-05441-1)

Additional file 2: Figure S2. Partial least-squares discriminant analysis model overview depicting the optimal number of components used to build the model (p1-p5). The Y-axis represents the cumulative fit (R2Y) and prediction (Q2Y) coefficients for each of the components (A). Validation of the model (permutation test, 500 permutations) to estimate R2Y and Q2Y significance. pR2Y and pQ2 are considered significant at $P < 0.05$ (B). Observation diagnostics was performed to detect outliers by plotting the score and orthogonal distances of each sample (red = control fish; blue = fish with low/medium infection degree; green = fish with high infection degree). No outliers were detected in this model (C) (PDF).

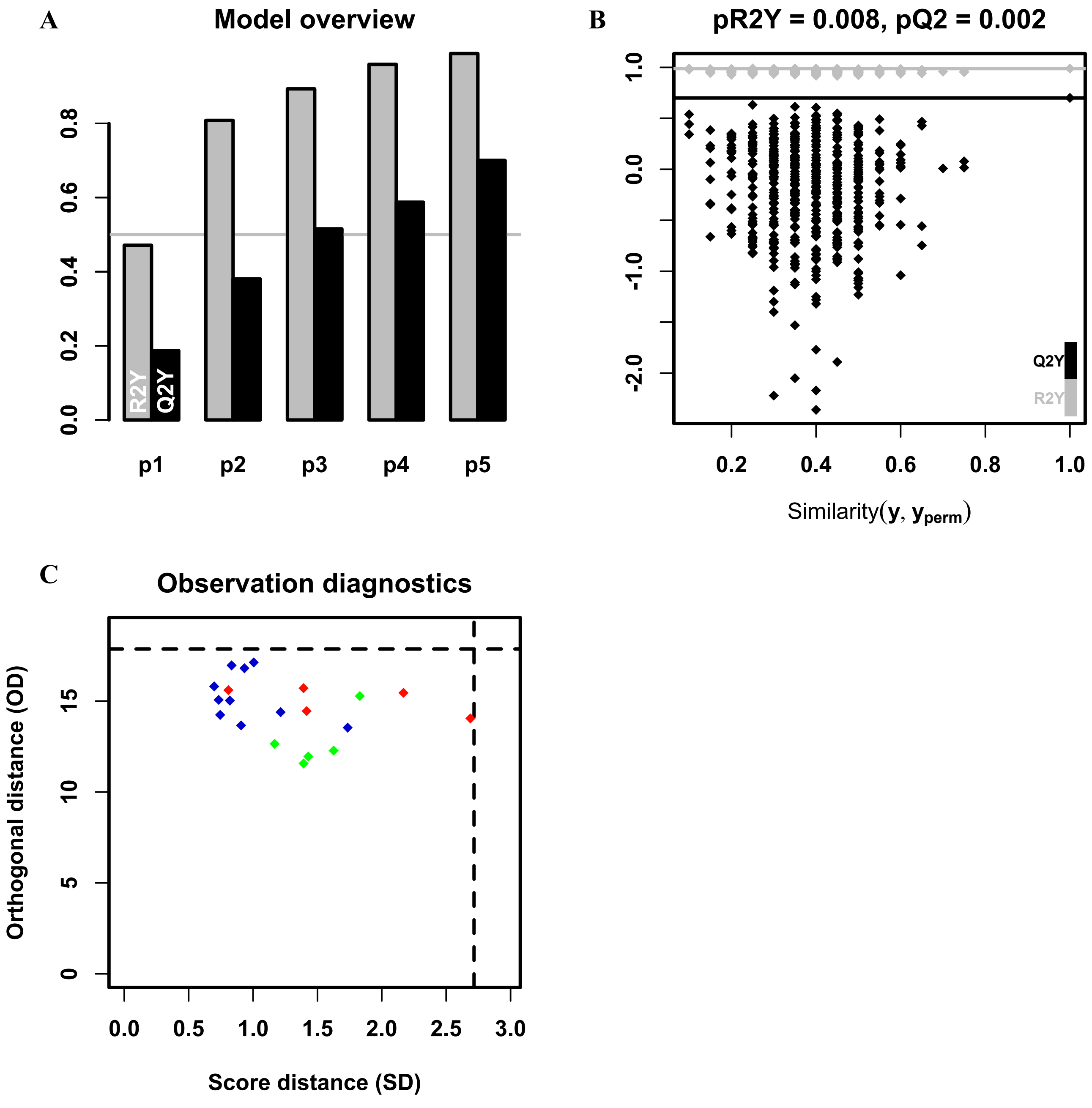

Supplement: Supplementary file 2 — Additional file 2: Figure S2. Partial least-squares discriminant analysis model overview depicting the optimal number of components used to build the model (p1-p5). The Y-axis represents the cumulative fit (R2Y) and prediction (Q2Y) coefficients for each of the components (A). Validation of the model (permutation test, 500 permutations) to estimate R2Y and Q2Y significance. pR2Y and pQ2 are considered significant at P < 0.05 (B). Observation diagnostics was performed to detect outliers by plotting the score and orthogonal distances of each sample (red = control fish; blue = fish with low/medium infection degree; green = fish with high infection degree). No outliers were detected in this model (C). [file 13071_2022_5441_MOESM2_ESM.pdf]
