# Supplementary material for: A bloody interaction: plasma proteomics reveals gilthead sea bream (Sparus aurata) impairment caused by Sparicotyle chrysophrii
Source: Parasit Vectors. 2022 Sep 10;15:322. doi: 10.1186/s13071-022-05441-1 (PMC9463799; doi:10.1186/s13071-022-05441-1)

Additional file 4: Figure S3. Plasma biotin values measured in control (C, *n* = 50) and *Sparicotyle chrysophrii* infected fish with a medium/low (M/L, *n* = 27) and high (H, *n* = 10) infection degree (A). Normalized protein abundance values of biotinidase (B) measured by proteomics in plasma samples of control (C, *n* = 5), medium/low (M/L, *n* = 10), and high (H, *n* = 5) infection groups. Values are represented as mean ± SEM and statistical differences among groups are noted with different letters (Kruskall-Wallis test, $p < 0.05$).

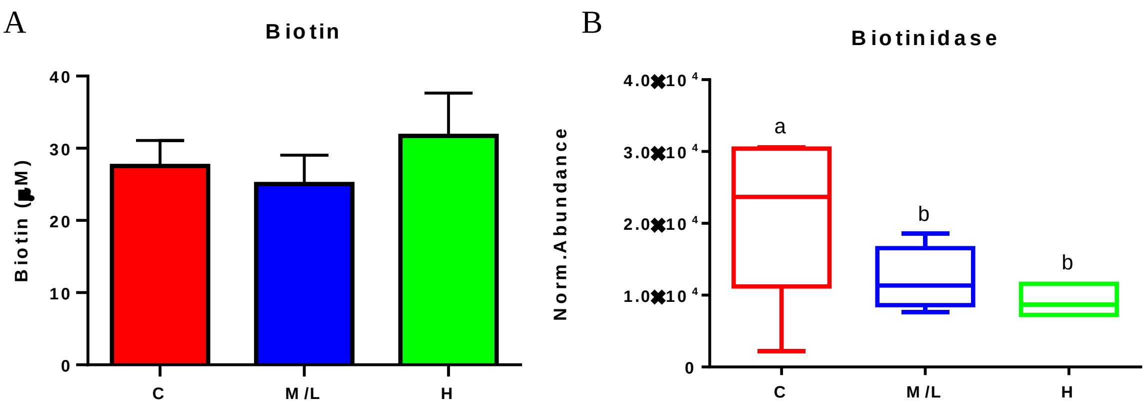

Supplement: Supplementary file 4 — Additional file 4: Figure S4. Plasma biotin values measured in control (C, n = 50) and Sparicotyle chrysophrii-infected fish with a medium/low (M/L, n = 31) and high (H, n = 16) infection degree (A). Normalised protein abundance values of biotinidase (B) measured by proteomics in plasma samples of control (C, n = 5), medium/low (M/L, n = 10), and high (H, n = 5) infection groups. Values are represented as mean ± SEM and statistical differences among groups are noted with different letters (Kruskall-Wallis test, P < 0.05). [file 13071_2022_5441_MOESM4_ESM.pdf]
